# Seventeen antibiotic-producing bacteria isolates found across four freshwater environments

**DOI:** 10.17912/micropub.biology.001353

**Published:** 2024-11-14

**Authors:** Caymen Hoffman, Edgar Caracoza, Kevin Kyaw, Kristina Blanke

**Affiliations:** 1 Biology, Beloit College, Beloit, Wisconsin, United States; 2 Pakula Biomedical Fellowship

## Abstract

Antibiotics are produced by microorganisms as defense mechanisms against bacteria and have treated bacterial infections for decades. Most of the current antibiotics are extracted from soil bacteria, and no new antibiotic class has been found in nearly 40 years. However, antibiotic-producing bacteria were discovered on tree bark, emphasizing that other environments should be explored for these bacteria. This research identified a new environment for antibiotic-producing bacteria–freshwater. Bacteria from freshwater sources in Wisconsin were cultured and screened against nine tester bacteria. All four water sources contained antibiotic-producing bacteria; therefore, freshwater environments should be further studied for novel antibiotic-producing bacteria.

**Figure 1. Isolates from four water sources produced antibiotics against Gram-negative, Gram-positive, and acid fast bacteria:**
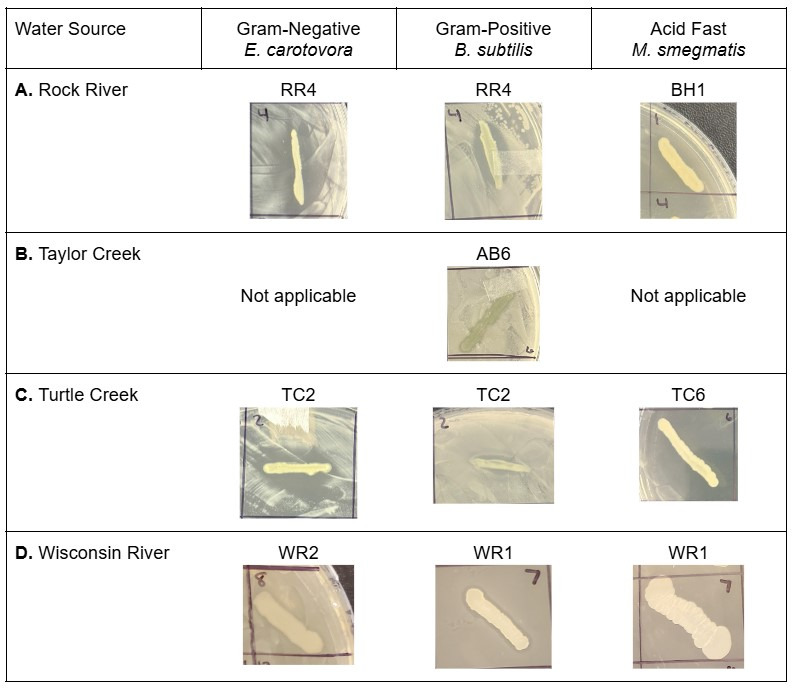
Zones of inhibition are shown around select isolates screened against *Erwinia carotovora* (Gram-negative bacteria), *Bacillus subtilis* (Gram-positive bacteria), and *Mycobacterium smegmatis* (acid fast bacteria). Each isolate is labeled based on its sampling location and the isolate number: AB = Avon Bottoms Wildlife Area, BH = Big Hill Park, RR = Rock River, TC = Turtle Creek, WR = Wisconsin River. **Panel A.** Rock River isolate RR4 produced antibiotics against *E. carotovora* and *B. subtilis* on potato dextrose agar (PDA). Rock River isolate BH1 produced antibiotics against *M. smegmatis* on 50% tryptic soy agar (TSA). **Panel B.** The one isolate from Taylor Creek within Avon Bottoms Wildlife Area produced antibiotics against *B. subtilis* on nutrient agar (NA), but did not produce antibiotics against *E. carotovora* or *M. smegmatis* (no zone of inhibition). **Panel C.** Turtle Creek isolate TC2 produced antibiotics against *E. carotovora* and *B. subtilis* on PDA. Turtle Creek isolate TC6 produced antibiotics against *M. smegmatis* on 50% TSA. **Panel D.** Wisconsin River isolate WR2 produced antibiotics against *E. carotovora* on lysogeny broth agar (LBA). Wisconsin River isolate WR1 produced antibiotics against *B. subtilis* and *M. smegmatis* on LBA.

## Description

The majority of current antibiotics were initially produced by soil bacteria, but a new class of antibiotics has not been discovered in almost 40 years (Allen et al., 1987) . However, other environments contain antibiotic-producing bacteria, such as tree bark (Hoffman & Blanke, 2024) . Additionally, recent studies found promising antibiotic-producing bacteria in hot springs (Kortam et al., 2023) , thermal springs (Alqahtani et al., 2023) , and freshwater (Shrestha et al., 2021) .

This research identified if antibiotic-producing bacteria exist in freshwater environments. Water was collected from two creeks (Taylor Creek and Turtle Creek) and two rivers (Rock River and Wisconsin River) in Central and Southern Wisconsin. Bacteria from the water sources included isolates that produced antibiotics against all nine tester bacteria. Additional novel environments, such as freshwater, could provide a solution to the current healthcare crisis where pathogenic bacteria are developing antibiotic resistance to currently used antibiotics and driving the cause for finding novel antibiotic-producing bacteria.

Three of the four water sources contained bacteria that produced antibiotics against Gram-positive, Gram-negative, and acid fast bacteria; however, Taylor Creek only contained one isolate that produced antibiotics against Gram-positive bacteria ( Figure 1 ). Most antibiotic activity was against Gram-positive bacteria with a total of 12 isolates across the four water systems (Table 1). There were seven isolates with antibiotic activity against Gram-negative and acid fast bacteria.

**Table 1.** Isolates from four water systems produced antibiotics against Gram-negative, Gram-positive, and acid fast tester bacteria. All four water systems had unique combinations of bacteria genera that inhibited tester bacterial growth. Each isolate with antibiotic activity was effective against 1-2 categories of tester bacteria.

**Table:** 

Water Source	Genera	Total Number of Isolates Tested	Number of Isolates with Antibiotic Activity	Gram- Negative	Gram- Positive	Acid Fast
Rock River	*Bacillus* *Pantoea* *Pseudomonas* *Rheinheimera*	36	6	2	4	3
Taylor Creek	*Chitinimonas*	6	1	0	1	0
Turtle Creek	*Bacillus* *Buttiauxella* *Calidifontibacillus* *Pantoea* *Rheinheimera*	33	6	4	4	2
Wisconsin River	*Chromobacterium* *Pseudomonas*	32	4	1	3	2

Seventeen antibiotic-producing isolates were found across the four freshwater environments (Table 1). Turtle Creek and Rock River samples had the highest number of antibiotic-producing bacteria, each containing six isolates. Turtle Creek and Rock River water samples were cultured on three media, with each media type cultivating two antibiotic-producing isolates per location. The most diverse genera were found in Turtle Creek, and two genera were only found in this water source ( *Buttiauxella* and *Calidifontibacillus)* . Three genera from Turtle Creek were shared with Rock River isolates ( *Bacillus, Pantoea,* and *Rheinheimera)* . Wisconsin River isolates shared one genus with Rock River isolates ( *Pseudomonas* ), while having one genus only found in the Wisconsin River ( *Chromobacterium* ). One genus ( *Chitinimonas* ) was only found in Taylor Creek. Genera diversity at the different sites could indicate the various levels of competition found in water. More competition could propel bacteria to produce antibiotics for an advantage in their environment. This is supported by the higher number of genera and percentage of isolates with antibiotic activity in the Rock River and Turtle Creek compared to the Wisconsin River.

The percentage of isolates with antibiotic activity based on the total number of isolates tested was similar (17-18%) for the Rock River, Taylor Creek, and Turtle Creek (Table 1). The Wisconsin River samples had 12.5% of isolates with antibiotic activity. The percentage of antibiotic activity in each water system indicates that freshwater contains a consistent number of antibiotic producers and is a valid source to identify antibiotic-producing bacteria.

Multiple samples were collected from three of the water sources and each had more than one isolate with antibiotic activity; however, only one sample was collected from Taylor Creek, which limits the number of bacteria tested for antibiotic-production. Therefore, Taylor Creek may contain more than one antibiotic-producing isolate and could include isolates effective against Gram-negative and acid fast bacteria. Most of the tested bacteria were from the original water samples, indicating that water should be filtered to concentrate the bacteria from each source before plating. Slight modifications to the protocol could increase the number of antibiotic-producing isolates discovered.

Several of the seventeen isolates identified from the water sources showed antibiotic activity against three or more tester bacteria. These promising isolates will be identified through 16S rRNA sequencing to verify the genus and species. Isolates will be further characterized through biochemical tests and the results will be compared to the known characteristics for the given genus and species.

In conclusion, seventeen antibiotic-producing isolates were found across four freshwater systems, indicating that water should be further studied for bacteria with antibiotic activity.

## Methods

Sample Collection and Preparation

Four water systems were sampled across Central and Southern Wisconsin, United States: Rock River (Beloit), Taylor Creek (Broadhead), Turtle Creek (Beloit), Wisconsin River (Wisconsin Dells). Water samples were collected in shallow depths at each water system between 7.5-31 cm below the surface. Only one 15 mL water sample was collected at each sampling site. Original water samples were plated to culture bacteria and 1 mL of water from each sample was used to create serial dilutions (Hernandez et al., 2018) . Bacteria were cultured on various media (LBA, NA, PDA, or 50% TSA) and incubated around 27°C for 48 hours.

Colony Selection

Colonies from the original sample and serial dilutions were selected for antibiotic activity screening based on variations in size, surface, color, form, margin, and elevation. Taylor Creek was only sampled once and six isolates were screened for antibiotic activity. The Rock River, Turtle Creek, and Wisconsin River were sampled at different locations along their water systems and 32-36 isolates from each water system were screened for antibiotic activity.

Antibiotic Activity Screening

Select isolates from the water samples were screened against nine tester bacteria ( *Acinetobacter baylyi, Enterobacter aerogenes, Erwinia carotovora, Escherichia coli, Pseudomonas putida, Bacillus subtilis, Enterococcus raffinosus, Staphylococcus epidermidis, Mycobacterium smegmatis* ) as previously described (Hoffman & Blanke, 2024) . The tester bacteria were categorized as Gram-negative, Gram-positive, or acid fast bacteria. Screens were conducted on various media (LBA, NA, PDA, or 50% TSA) and incubated around 27°C for 48 hours. Visible zones of inhibition identified that the isolate produced antibiotics against a particular tester bacteria.

Verification of Bacteria Genera through Sequencing and BLASTn

Isolates that showed antibiotic production against the tester bacteria were processed through colony PCR and gel electrophoresis, then each genus was identified with 16S rRNA sequencing (Hernandez et al., 2018) . Sequencing was performed by Eurofins Genomics (Louisville, KY), and the generated sequencing text was run through BLASTn (courtesy of the National Library of Medicine).
